# Systematic study on date palm seeds (***Phoenix*** ***dactylifera*** L.) extraction optimisation using natural deep eutectic solvents and ultrasound technique

**DOI:** 10.1038/s41598-024-67416-9

**Published:** 2024-07-18

**Authors:** Alanood A. Alfaleh, Heba A. Sindi

**Affiliations:** 1 Department of Environmental Sciences, Faculty of Environmental Sciences, King Abdulaziz University, Jeddah, Saudi Arabia; 2 Food and Nutrition Department, Faculty of Human Sciences and Design, King Abdulaziz University, Jeddah, Saudi Arabia

**Keywords:** Natural deep eutectic solvents, Date seeds, Polyphenols, Ultrasound, Conventional solvents, Biophysics, Environmental sciences

## Abstract

Natural deep eutectic solvents (NADES) are emerging, environment-friendly solvents that have garnered attention for their application in extracting phenolic compounds. This study investigated the effects of four synthetic NADES on polyphenols extracted from date seeds (DS) using choline chloride (ChCl) as a hydrogen-bond acceptor and lactic acid (La), citric acid (Citri), glycerol (Gly), and fructose (Fruc) as hydrogen-bond donors, in comparison with DS extracts extracted by conventional solvents (water, 70% methanol, and 70% ethanol). The antioxidant activity (DPPH), total phenolic content (TPC) and 6 phenolic compounds were determined using HPLC. The results showed that the ChCl–La and ChCl–Citri systems exhibited a high extraction efficiency regarding TPC, and DPPH in the DS extracts extracted by NADES compare to those DS extracts extracted with conventional solvents (*p* ˂ 0.001). HPLC results demonstrated that DS extracted by ChCl–La contained all measured phenolic compounds. Also gallic acid and catechin were the major compounds identified in the DS extracts. In addition DS extracted by ChCl–Citri and ChCl–Gly had the highest concentration of catechin. In conclusion, combining NADES is a promising and environment-friendly alternative to the conventional solvent extraction of phenolic compounds from DS.

## Introduction

Selecting the right extraction method is crucial for extraction operations^1^. Extraction is the first and most important step in obtaining phytochemicals from plant sources^2^. Several extraction methods have been utilised to obtain beneficial chemicals from plant matrices, but they have limitations such as the use of large quantities of hazardous solvents and long duration of procedures. Organic solvents are commonly used due to their strong potential to form hydrogen bonds with phenolic chemicals^3–5^. However, these solvents are unable to meet both environmental and economic needs.

Recently, the development and implementation of environmental-friendly technologies have gained popularity because they boost extraction efficiency while reducing solute component degradation^1,6^. Deep eutectic solvents (DES) are non-aqueous solvents formed from ionic liquids containing a hydrogen-bond acceptor and a hydrogen-bond donor component, and they are characterised by their lower melting temperatures according to Abbott et al.^7^

Thus, new solvents are being investigated for DES to extract phenolic chemicals, and natural deep eutectic solvents (NADES) have been used to replace toxic solvents for green consumerism^6^. Different types of NADES have shown tremendous potential in various applications due to their non-toxicity, sustainability, solubility, and environmental friendliness^8,9^. NADES are a suitable, eco-friendly medium for extracting bioactive compounds and has many advantages including easy preparation and use, inexpensive material costs, and low vapour pressure^10–13^.

Notably, there has been an increasing attention on agricultural residues such as seeds, peels, and pomace of plants and the extraction of their bioactive phenolic compounds^14^. These compounds exhibit health benefits such as the lowering of the risk of cancer, cardiovascular disease, and diabetes, and potent functions including anti-bacterial and anti-inflammatory properties, making them valuable in the food, chemical, and pharmaceutical in food, chemical, and pharmaceutical industries^15–17^.

Date seeds (DS) are sub-products of date palm (*Phoenix* *dactylifera*) fruits. They contribute to a considerably large amount of food waste, reaching approximately 1 million tons annually, which classifies them as an environmental problem^18,19^. Jabeen et al.^18^ reported that > 8 million tons of dates are produced annually. The ineffective use of this sub-product for human food constitutes a considerable economic loss because of the nutraceutical properties of DS, which are mediated by high levels of dietary fibre, protein, fat, minerals, antioxidants, and bioactive compounds such as polyphenols^20,21^. DS has been recognized to have beneficial polyphenolic compounds according to Al-Farsi and Lee^22^. Several studies have exhibited significant antimicrobial, anticancer, antioxidant activities and total phenolic contents quantified and identified the phenolic compounds in different kinds of DS^23–26^ Additionally, DS compared to date fruit demonstrated higher total phenolic contents which consider as an extraordinary source of phenolic compounds^27^. DS contain bioactive compounds, such as polyphenols, especially phenolic acids, and flavonoids it is present in large quantities in DS^23,28^. Alkhoori et al.^29^ demonstrated that date seeds contain a variety of polyphenols, including *p*-coumaric acid, gallic acid, syringic acid, caffeic acid, chlorogenic acid, rutin, quercetin, and catechin are prominent polyphenols found in date seeds.

According to Choi et al.^30^, many primary metabolites of plants such as choline chloride (ChCl), sugars, and carboxylic acid are possible components of normal ionic liquids and DES; these compounds can form a DES-like liquid when mixed in certain ratios, resulting in the generation of a novel type of DESs called NADES.

Rutin is one of the flavonoids that are more soluble in NADES than in water^30,31^. To extract catechin, anthocyanins, and quercetin-3-*O*-glucoside from grape skins, Bubalo et al.^32^ assessed five NADES and revealed that the most efficient extraction solvent was NADES containing ChCl:oxalic acid (1:1) and 25% water. Dai et al.^33^ extracted phenolic compounds from safflower using seven NADES of varying polarity and found that the NADES yielded higher extraction compared with the conventional solvents.

However, far too little attention has been paid to studies and investigated using NADES of the polyphenol’s extraction from date palm seeds. Therefore, the aim of this study evaluating the polyphenols from date palm seeds (*Phoenix* *dactylifera* L.) extracts obtained by NADES with ultrasound-assisted extraction technique.

## Materials and methods

### Materials

Khalas Date palm seeds (*Phoenix* *dactylifera* L., family: *Arecaceae*) cultivated in 2021 were selected from three random date factories (Al-Khammash dates factory, Al-Kharj, KSA), (Nadeed Alwashm factory for dates, Riyadh, KSA), (Al-Muallim dates, Al-Qassim, KSA), factories subject to the supervision of the Saudi Ministry of Environment, Water and Agriculture, which relies on local and international rules and regulations, as the Kingdom is one of the most important and largest producers and exporters of palm dates in the world.

Choline chloride (> 98.0%), citric acid (Citri, > 98.0%), glycerol (Gly, > 99.0%), fructose (Fruc, 99.0%), gallic acid, 2,2-diphenyl-1-picrylhydrazyl (DPPH), Folin–Ciocalteu’s reagent, phosphate buffer (pH 7), high-performance liquid chromatography-grade solvents (methanol [MeOH] and ethanol [EthOH]), and sodium carbonate were obtained from Sigma Aldrich Chemical (St. Louis, MO, USA). Lactic acid (La, > 98.0%) was obtained from PanReac AppliChem (Barcelona, Spain), and the Nile red (NR) dye was from Solarbio Science & Technology Co., Ltd. (Beijing, China).

### Methods

#### Preparation of NADES

NADES were prepared according to the methodology of Chanioti and Tzia^1^ with minor modifications. Some of the modifications involve mixing ChCl as a hydrogen bond acceptor with La, Citri, Gly, or Fruc as hydrogen bond donors in molar ratios (Table 1) by placing them in a water bath at 80 ± 5 °C for 1 h. Following this, the mixtures were transferred onto a magnetic stirrer hot plate and subjected to heating and stirring in the same condition for 1–6 h until the mixture became clear. All NADESs were diluted with 20% (v/v) water and kept in sealed containers at room temperature (20 °C).

**Table 1:** List of abbreviations and the composition of natural deep eutectic solvents.

Code	Composition	Molar ratio	Water content (%)
ChCl–La	Choline chloride:lactic acid	1:2	20%
ChCl–Citri	Choline chloride:citric acid	2:1	
ChCl–Gly	Choline chloride:glycerol	1:2	
ChCl–Fruc	Choline chloride:fructose	1:2	

#### Preparation of DS powder

DS were randomly homogenised, washed, and dried in an oven for 12 h at 50 °C. The seeds were ground using a heavy-duty grinder (Al Saif Professional Coffee Grinder Size: 800 g, 2400 W) until 0.5-mm size particles were obtained.

#### Ultrasound-assisted solvent extraction

DS powder was mixed in a ratio of 1:10 (weight:volume) with seven different solvents, namely water, 70% MeOH, 70% EthOH, or one of four mixtures of NADESs prepared as mentioned previously, and the mixture was homogenised at a speed of 800 rpm for 3 min using a homogeniser. The extracts were then transferred to the ultrasound bath (HumanLab Instrument Co., Korea) at 40 °C for 30 min. Finally, the supernatant extracts were centrifuged at 2100× g for 15 min and kept at − 20 °C until use.

#### Physicochemical properties of NADES (namely pH, viscosity, and polarity)

The pH was measured at room temperature using a pH meter (Jenway 3510 Standard Digital pH Meter, Bibby Scientific Ltd., UK). The Brοοkfield rheometer (Brookfield Engineering Laboratories Inc., Stoughton, MA) using strain S62 at the speed of 30 rpm at 25 °C was used to measure the viscosity. Polarity was measured by the Reichardt and Dimroth’s scale^34^ using 10 μL of the NR dye (1 mg/mL EthOH), which was added to each NADES and the conventional solvent (70% EthOH, 70% MeOH, and water) as a solvatochromic probe to find the maximum absorption wavelength ($\uplambda$max) of the NR dye mixtures at 400–700 nm using the ultraviolet (UV)–visible (vis) spectrophotometer (Model UV-3600, Shimadzu, Kyoto, Japan) at room temperature. The NR polarity parameter was calculated using the following equation:

$${\text{ENR}}\;\left( {{\text{kcal}}/{\text{mol}}} \right) = 28591/\uplambda \;{\text{abs}}\;\left( {{\text{nm}}} \right)$$

where ENR denotes electron transition in NR and $\uplambda$ abs is the maximum absorbance.

#### Fourier-transformed infrared (FTIR) analysis

FTIR is an analytical technique used to identify organic, polymeric, and in some cases, inorganic materials. The FTIR analysis method uses infrared light to scan the test samples and analyse their chemical properties.

FTIR analysis was performed for the optimal NADES (namely ChCl–La, ChCl–Citri ChCl–Citri, ChCl–Gly, ChCl–Fruct) and their individual components (ChCl, La, Citri, Gly and Fruc). The analysis was carried out at wavenumber range of 4000–400 cm^−1^ using Nicolet iS10 FTIR Spectrometer (Thermo Fisher Scientific Inc., USA)**.**

#### Determination of total phenolic content (TPC) using the Folin–Ciocalteu method

To determine the TPC of extracts, Folin–Ciocalteu’s reagent was used according to the method of Lanjekar et al.^35^ with minor modifications.

The quantification of TPC was based on the standard curve created using stock solutions of gallic acid (1 mg/1 mL w/v) at different concentrations. The solutions were prepared by twofold serial dilutions and analysed using a GENESYS 10S UV–Visible Spectrophotometer, and the regression coefficient (R^2^) of 0.998 indicated good correspondence. The samples were diluted using phosphate buffer (pH 7), and 100 μL of DS extracts were mixed with 200 μL of Folin–Ciocalteu’s reagent (10%; v/v). After 5 min, 2000 μL of 7.5% sodium carbonate solution was added to the reaction mixture, followed by 2 h incubation at room temperature in the dark. The absorbance values of all the samples were measured at 765 nm using UV–vis spectrophotometer.

TPC of the extracts were analysed in triplicates, and the results are expressed as mg of gallic acid equivalents (GAE) per g of dry weight (dw).

TPC (mg GAE/g dw) was calculated using the following formula:

$${\text{TPC}} = {\text{GAE}} \times {\text{V}} \times {\text{D}}/{\text{W}}$$

where GAE is gallic acid equivalent (mg/mL), D is the dilution factor, V is the volume of extraction solvent (mL), and W is the weight (g) of DS.

#### Antioxidant activity determination by the DPPH radical scavenging assay

The antioxidant activities of the DS extracts were determined by performing the DPPH radical scavenging assay according to the methodology of Lanjekar et al.^35^ with some adjustments. First, 100 μL of the DS extract or the control solvent and 1500 μL of the DPPH solution were mixed well by vortexing. Next, the mixtures were incubated for 1 h at room temperature in the dark. Finally, all the samples were diluted using the phosphate buffer (pH 7) and assessed at 517 nm for DPPH radical scavenging activity.

#### HPLC–UV/VIS analysis

According to the methods of Lanjekar et al.^35^ reversed-phase (RP) high performance liquid chromatograph HPLC was used for the identification and quantification of polyphenols in seven DS extracts using system (Shimadzu, Kyoto, Japan) samples were measured duplicate. For the analysis the separation C18 column (250 × 4.6 mm × 5 μm) (MZ Analysentechnik, Mainz, Germany), UV–VIS detector, quaternary pump. The column was incubated at 40 °C and the autosampler cooled to 4 °C. The samples (20 μL) were injected. The mobile phase consisted of a combination of solvent A (acetonitrile) and solvent B (distilled water/acetic acid, 99:1, v/v, pH 2.30 ± 0.1) at a flow rate of 1 mL/min. The gradient program used for the separation of target bioactive compounds was 20% A (5 min), 80% A (10 min), 20% A (5 min). The detector was set at 280 nm for gallic acid, catechin, syringic acid, *p*-coumaric acid, and 370 nm for rutin, quercetin.

The standard curve was used to calculate the concentration of the standards polyphenols. The concentrations of standard solutions 25, 50, and 100 ppm were plotted. After injecting the solutions, the area under the curve was noted.

### Statistical analyses

Analyses were performed using the International Business Machines Statistical Package for Social Sciences Statistics software version 27. All experimental data are represented as the mean of triplicates ± standard deviation. Additionally, one-way analysis of variance, multivariate correlation analysis based on Pearson’s correlation, and least significant difference test were performed. Differences at the *p* value of < 0.05 were considered statistically significant.

## Results and discussion

### Determination of the physicochemical properties (pH, viscosity, and polarity) of NADES

**Figure 1:**
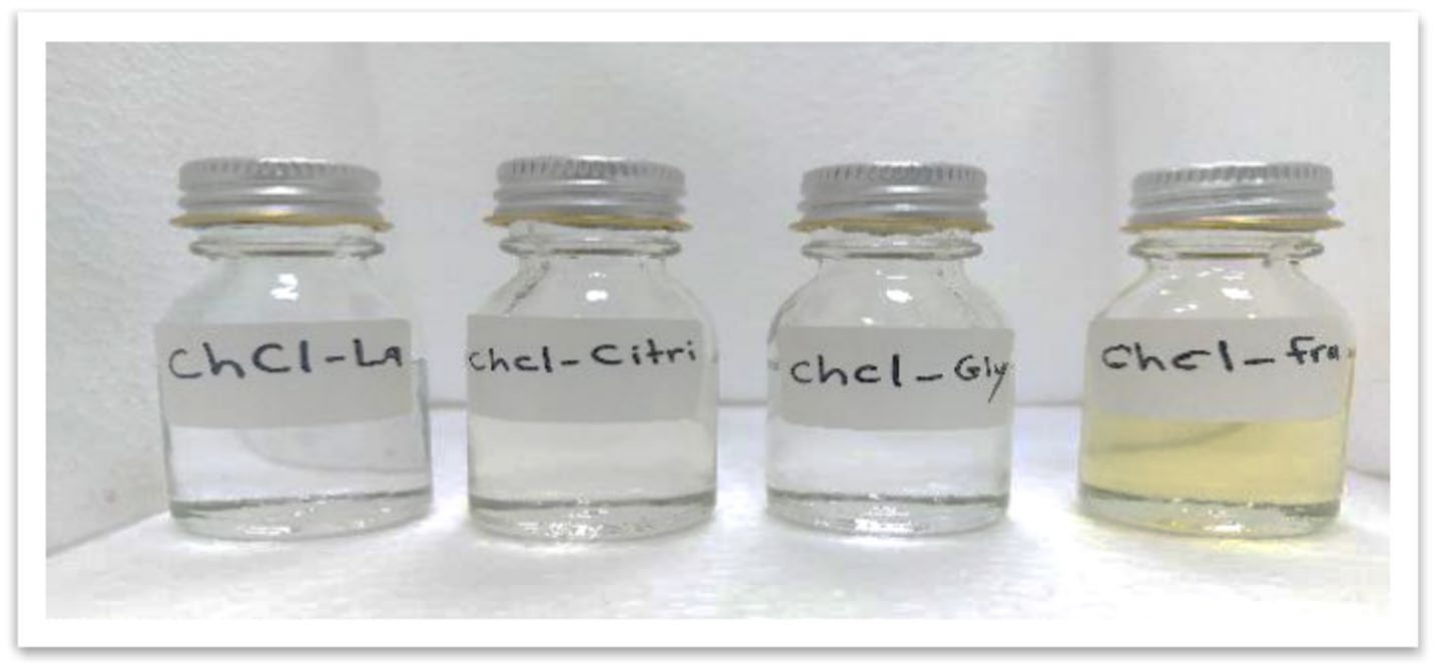
Appearance of natural deep eutectic solvents, starting from the left: choline chloride:lactic acid (ChCl–La), choline chloride:citric acid (ChCl–Citri), choline chloride:glycerol (ChCl–Gly), and choline chloride:fructose (ChCl–Fruc).

All prepared NADES are shown in Fig. 1. Notably, the acid-based (ChCl–La, ChCl–Citri) and sugar alcohol-based solvents (ChCl–Gly) are colourless, whereas a sugar-based solvent (ChCl–Fruc) is yellow, consistent with the reports of Chanioti and Tzia^1^ and Airouyuwa et al.^36^.

The physicochemical properties of all solvents are presented in Table 2. Conventional solvents, including water, 70% MeOH, and 70% EthOH, showed neutral pH. The lowest pH (acidic) values were observed for ChCl–La, ChCl–Citri, ChCl–Fruc, and ChCl–Gly. The pH values were mainly influenced by the constituents, with NADES containing a hydrogen bond donor contributing to the acidic pH value^37,38^.

**Table 2:** Physical and chemical properties of natural deep eutectic solvents (NADES).

Type of solvent	pH	Viscosity (30 rpm) (cP)	E_NR_ (kcal/mol^−1^)
Conventional solvents			
Water	7.16 ± 0.30^a^	0.87 ± 0.07^e^	43.17 ± 0.16f.
70% MeOH	6.40 ± 0.43^b^	0.94 ± 0.04^e^	52.26 ± 0.35^a^
70% EthOH	6.13 ± 0.25^b^	1.13 ± 0.15^e^	50.88 ± 0.18^b^
NADES			
ChCl–La	0.86 ± 0.04f.	18.38 ± 0.6^d^	45.54 ± 0.32^e^
ChCl–Citr	1.36 ± 0.40^e^	279.69 ± 10.64^a^	44.96 ± 0.60^e^
ChCl–Gly	5.57 ± 0.51^c^	41.94 ± 1.01^c^	50.03 ± 0.25^c^
ChCl–Fruc	4.74 ± 0.83^d^	236.56 ± 1.45^b^	47.09 ± 0.75^d^

Values are mean (*n* = 3) ± standard deviation. Different alphabet letters in the same column indicate significant differences (*p* < 0.05).

Previous studies have established a correlation between the pH of ChCl-based NADES and the presence of a hydrogen bond donor with an acidic molecule, followed by a sugar-based molecule and then a sugar-alcohol-based molecule^37–39^. Moreover, Skulcova et al.^40^ reported that the pH of ChCl–Gly with a molar ratio of 1:2 (around 4.9) was higher than that of ChCl with malonic acid with a molar ratio of 1:1 (around 2.4).

Viscosity is the most prominent characteristic of NADES and is one of the obstacles in handling such solvents because viscosity limits the transfer of compounds from the solid matrix to the liquid solution, thereby interfering with decantation, filtration, and dissolution^6,41^. The dilution of the ChCl-based NADES using water is one of the best approaches to overcome challenges posed by hydrogen bond formation between ion–water and hydrogen bond donor–water interactions^42,43^.

The viscosity of the ChCl-based NADES was measured after 20% dilution with water and expressed in centipoise (cP) units. The value can vary owing to the formation of a spacious hydrogen bond network, and this phenomenon is observed in most ChCl-based NADES with high viscosity at room temperature^44^.

No significant differences were observed between the conventional solvents and NADES in terms of the degree of viscosity. However, the degree of viscosity of NADES is greatly influenced by the nature of hydrogen bond donors in terms of being liquid or solid at room temperature. Therefore, the lower viscosities of ChCl–La and ChCl–Gly than those of ChCl–Citri and ChCl–Fruc could be attributed to La and Gly being liquids and Citri and Fruc being solids at room temperature. The hydrogen bond network formed within the NADES may affect the degree of viscosity. Moreover, the longer chain present in the NADES and the molar ratio between the hydrogen bond donor and acceptor may affect NADES viscosity^45^.

Polarity, which can be determined using the NR dye as a solvatochromatic probe, was expressed using Reichardt and Dimroth’s normalised (E_T_) scale, and the molar transition energy was denoted as E_NR_ when NR was used. The values indicated an inverse relationship; that is, the polarity increased when E_NR_ (kcal/mol) decreased. Table 2 presents the polarity of all solvents. These values ranged from 42.84 ± 0.72 kcal/mol to 52.23 ± 0.40 kcal/mol, and the highest value was observed for water and the lowest value for 70% MeOH. Although the acid-based NADES showed the highest polarity, the polarities of ChCl–La (45.54 ± 0.32 kcal/mol) and ChCl–Citr (44.96 ± 0.60 kcal/mol) were not significantly different.

Furthermore, the lowest polarity values were observed for sugar-based ChCl–Fruc (47.09 ± 0.75 kcal/mol) and sugar alcohol-based ChCl–Gly (50.03 ± 0.25 kcal/mol). Similarly, Fuad et al.^39^ reported the polarities of 20 NADES, which ranged from 44.81 to 58.58 kcal/mol, and acid-based NADES were the most polar, with E_NR_ values ranging from 44.81 to 48.3 kcal/mol. The E_NR_ values of sugar- and sugar-alcohol-based NADES were between 49.72 and 50.69 kcal/mol, whereas the alcohol-based NADES exhibited the lowest polarity (49.55–58.58 kcal/mol). Additionally, Fuad et al.^39^ assumed that the polarity of NADES decreased upon its dilution with water, whereas it increased with the increasing number of hydrogen bonds. Generally, molar ratios between hydrogen bond donors and acceptors affect the polarity value.

### FTIR analysis

**Figure 2:**
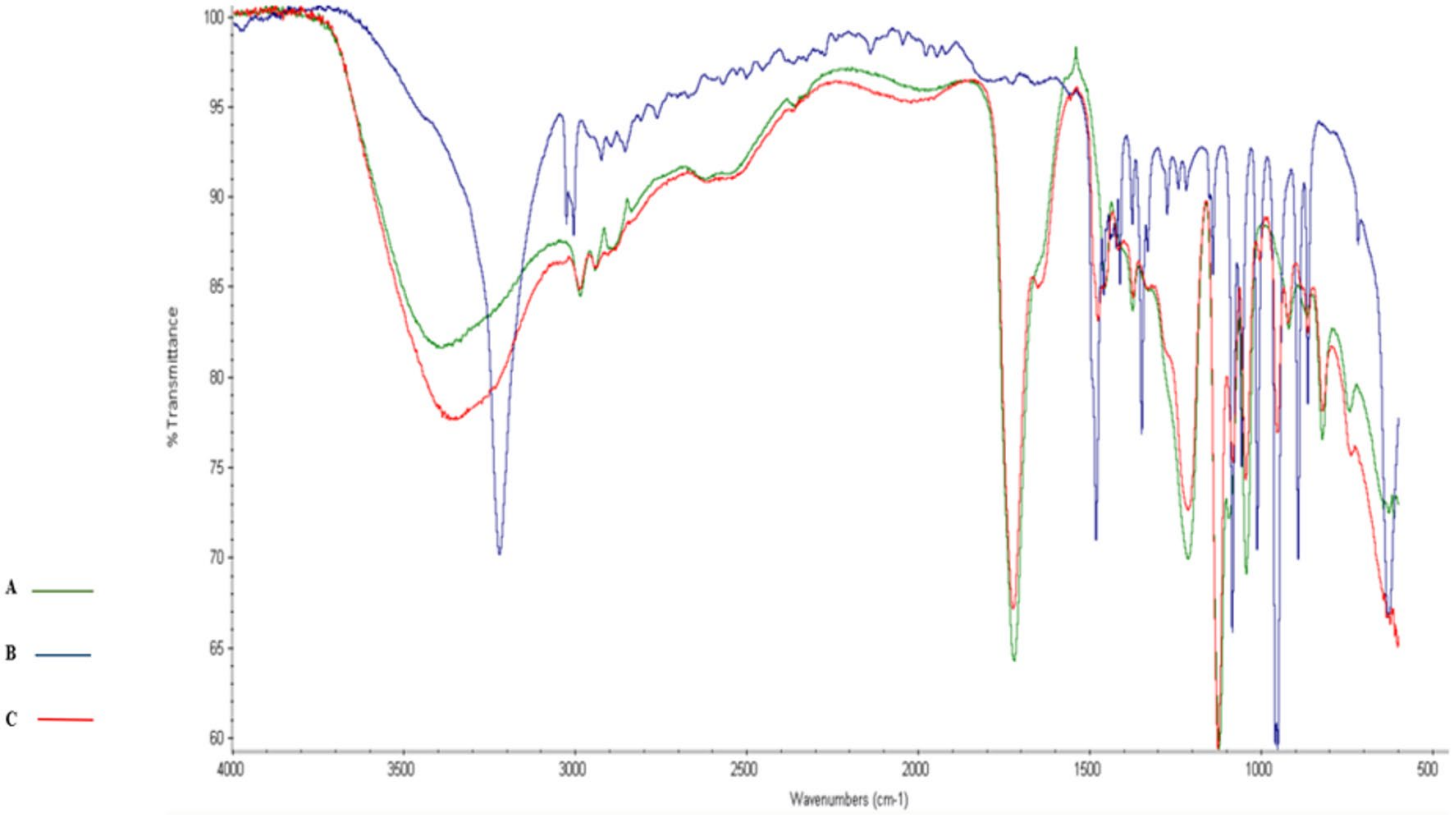
Fourier-transformed infrared spectra of the natural deep eutectic solvent (NADES) choline chloride (ChCl)–lactic acid (La) and their individual components: (A) La; (B) ChCl; and (C) NADES ChCl–La.

The FTIR spectra of La, ChCl, and ChCl–La with a molar ratio of 1:2 were examined at room temperature. The FTIR spectra of ChCl–La and their individual compounds are presented in Fig. 2. Characteristic absorption bands for the functional groups of La were observed at 3387 cm^−1^ (broad band for OH), 1720 cm^−1^ (C=O), 2986 cm^−1^ (C–H stretching), and 1455 cm^−1^ (C–H bending) in addition to the bands at 1120 cm^−1^ and 1043 cm^−1^ (C–O)^46,47^. The functional groups of ChCl showed sharp bands for OH at 3221 cm^−1^^46^, CH_3_ bending at 1481 cm^−1^, and C-N at 952 cm^−1^^48^. Furthermore, we observed a little shift in several absorption bands because of H-bond interactions between the functional groups of ChCl–La^49^. For instance, large broadness was observed for OH and N–H bands at 3600–2400 cm^−1^ and 1640 cm^−1^ in ChCl–La. Moreover, the peak for C-H bending in CH or CH_3_ shifted from 1455 to 1476 cm^−1^ in ChCl–La^50^.

**Figure 3:**
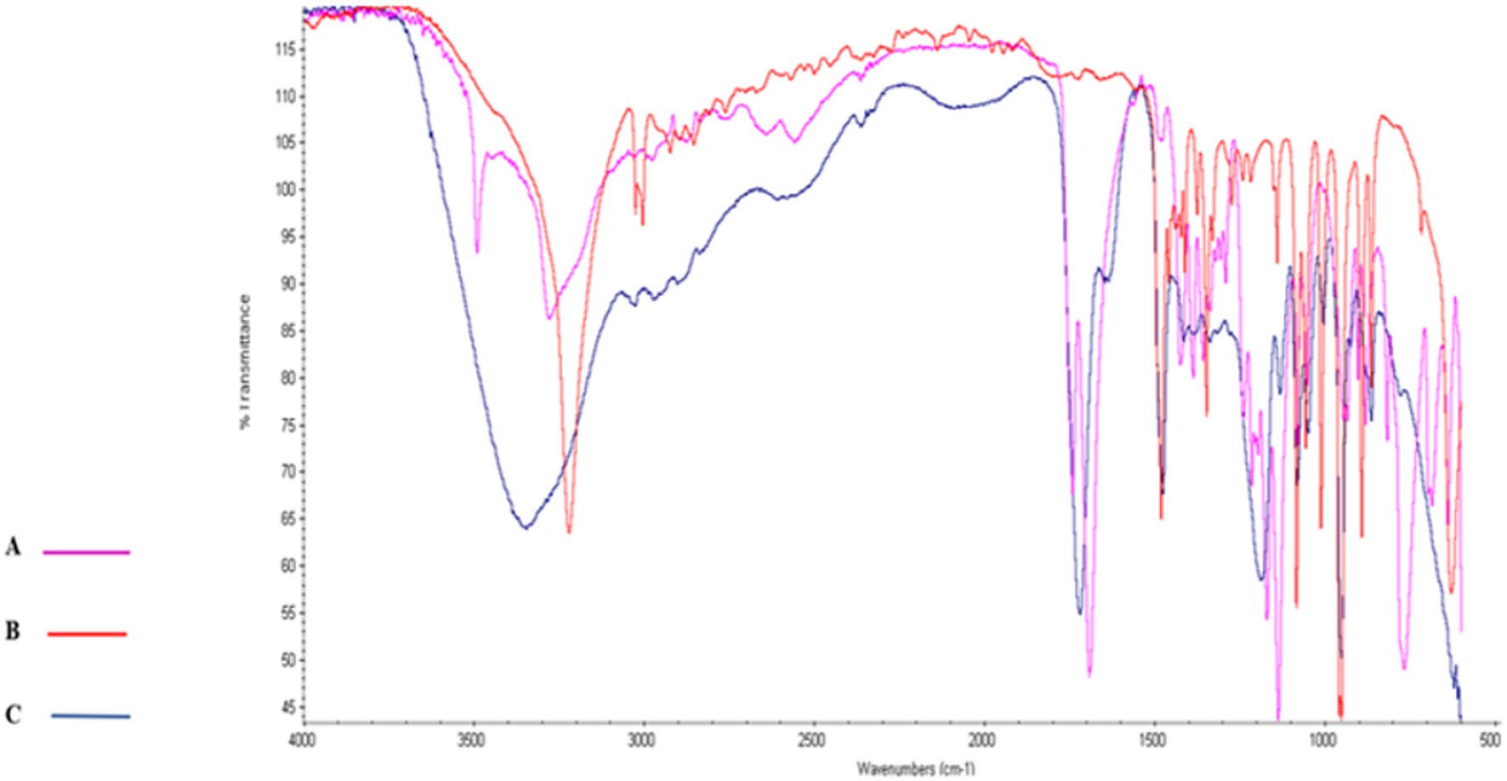
Fourier-transformed infrared spectra of the natural deep eutectic solvent (NADES) choline chloride (ChCl)–citric acid (Citri) and their individual components: (A) Citri; (B) ChCl; and (C) NADES ChCl–Citri.

The FTIR spectra of Citri, ChCl, and ChCl–Citri with a molar ratio of 2:1 were examined at room temperature. The three FTIR spectra illustrated in Fig. 3 showed several absorption band shifts because of H-bond interactions between the functional groups of the Citri and ChCl mixture. A broad band was observed at 3346 cm^−1^ in ChCl–Citri in addition to the downward shift of the carbonyl group stretching vibration from 1742 cm^−1^ and 1693 cm^−1^ to 1719 cm^−1^^50^.

**Figure 4:**
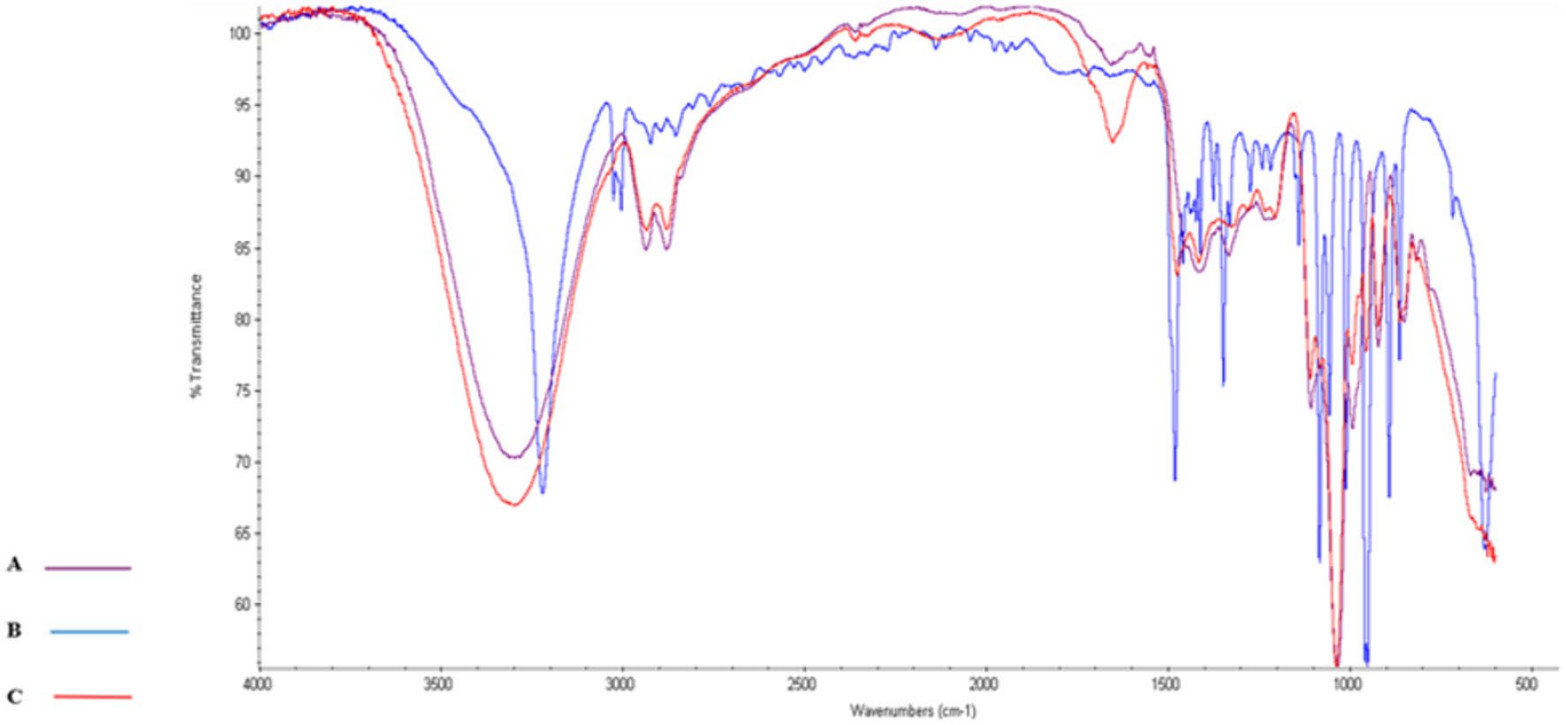
Fourier-transformed infrared spectra of the natural deep eutectic solvent (NADES) choline chloride (ChCl)–glycerol (Gly) and their individual components: (A) glycerol; (B) ChCl; and (C) NADES ChCl–Gly.

The FTIR spectra for the third NADES solvent that consists of ChCl–Gly were illustrated in Fig. 4 To compare the IR spectrum of the mixture with single compounds, the upward shift of dNH from 1481 choline chloride to 1653 in ChCl–Gly cm^−1^ refer to the appearance of H-bond between the glycerol and choline chloride^42^.

**Figure 5:**
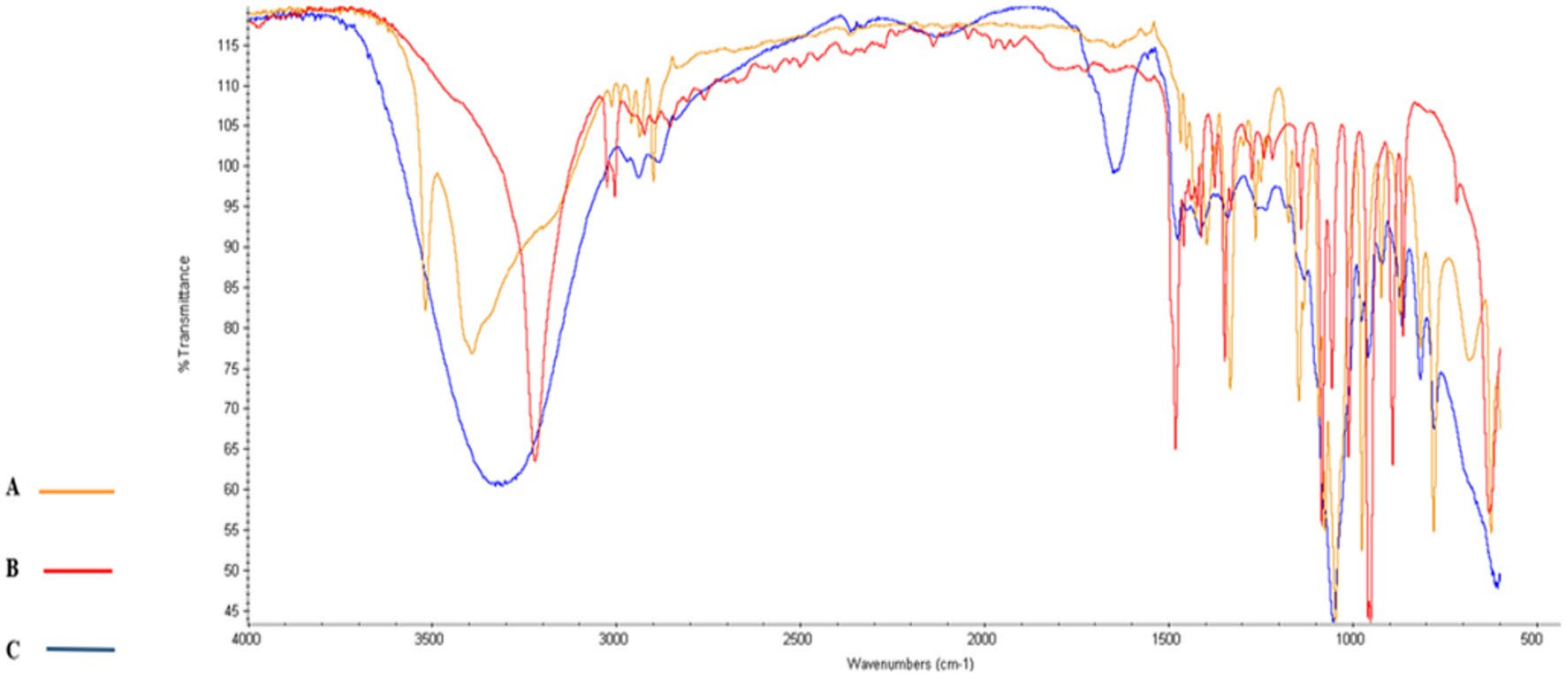
Fourier-transformed infrared spectra of the natural deep eutectic solvent (NADES) choline chloride (ChCl)–fructose (Fruc) and their individual components: (A) fructose; (B) ChCl; and (C) NADES ChCl–Fruc.

Figure 5 shows the IR spectra for fructose, choline chloride and their mixture (ChCl–Fruc). It is clear that a hydrogen bond was formed between fructose and choline chloride from the appearance of the broad band at 3329 cm^−1^ for the OH group in ChCl–Fruc. Another evidence for the formation of H-bond is the upward shift of dNH from 1481 (choline chloride) to 1652 (ChCl–Fruc) cm^−1^^42^.

### TPC and antioxidant activity of DS extracts

**Figure 6:**
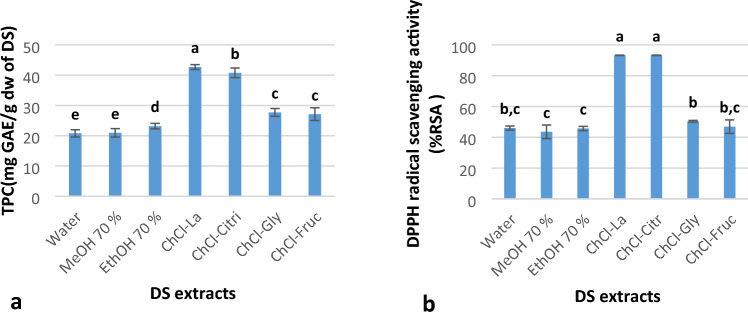
(**a**) Total phenolic content and (**b**) antioxidant activity of date seed (DS) extracted using conventional solvents and natural deep eutectic solvents (NADESs). The results are presented as mean (*n* = 3) ± standard deviation. MeOH, methanol; TPC, total phenolic content; EthOH, ethanol; La, lactic acid; ChCl, choline chloride; Citri, citric acid; Gly, glycerol; Fruc, fructose; GAE, gallic acid equivalent; RSA; radical scavenging activities and DPPH, 2,2-diphenyl-1-picrylhydrazyl.

The results of the TPC content of DS extracts implied the extraction performances of NADES and conventional solvents (Fig. 6). The results showed that the ChCI–La extract contained the highest TPC (42.68 ± 3.5 mg GAE/g dw), followed by ChCl–Citri (40.79 ± 9.6 mg GAE/g dw), compared with the conventional solvent containing TPC ranging from 23.16 ± 7.7 mg to 20.78 ± 4.8 mg GAE/g dw.

Figure 6 shows the percentage of the antioxidant activity of DS extracts using conventional solvents and NADES. ChCI–La and ChCl–Citri exhibited the highest radical scavenging activity (% RSA), whereas that of the conventional solvents converged with the ChCl–Fruc activity.

Phenolic compounds are extracted from different natural sources by the solid–liquid extraction method. Extracting phenolic compounds using NADES includes several key phenomena such as the transfer of mass from the solid phase to the liquid phase, the dissolving of phenolic compounds in NADES facilitated by their comparable polarities, and the occurrence of chemical interactions between NADES and phenolic compounds^44^.

Zannou and Koca^51^ and El Kantar et al.^52^ have reported that compared with the polarities of molecules in organic solvents (MeOH or EthOH), the higher polarities of molecules in NADES increase the extraction of polyphenols from the plant matrix because of the hydrophilic properties of NADES. Apostolakis et al.^53^ examined the potential of water/glycerol mixtures as a safe and efficient method for extracting polyphenols from olive leaves. They compared this approach to the use of water/ethanol mixtures and found that the polarity of the solvent may play a role in the extraction of phenolic compounds from the plant matrix. Specifically, they observed that highly polar polyphenols from olive leaves exhibited a high extraction rate when using a highly polar solvent such as water/glycerol. In contrast, less polar or non-polar polyphenols showed a lower extraction rate when using less polar solvents like water/ethanol. Several investigations have found that extracts generated from different plant materials employing similar NADES exhibit greater radical scavenging activities compared to aqueous, methanolic, and ethanolic extracts^38,39,54,55^. The results of current study align with the findings of Airouyuwa et al.^36^, who observed a notable increase in antioxidant activity in DS extracts when utilizing carboxylic acid as hydrogen bond donor in NADES at the similar ratio employed in our present study (1:2). In addition, a study conducted by Chanioti and Tzia^1^ found that olive pomace extracts prepared by different NADES (such as choline chloride:lactic acid, choline chloride:citric acid, and choline chloride:glycerol) with assisted ultrasound technology at a temperature of 40 °C exhibit significantly higher antioxidant activity compared to the water extract. The significant antioxidant activity of NADES extracts has been linked to their efficient recovery of bioactive compounds, according to several studies^35,56,57^. In general, according Fuad et al.^39^ the antioxidant capacity of NADES extracts tends to increase consistently with the quantity of isolated components. However, antioxidant activity might be indirectly influenced by factors such as viscosity, polarity of NADES and its molar ratio. In addition, the use of water enhances the antioxidant efficacy of the NADES extract. The enhanced solubility of the antioxidant chemicals is the cause, which is a result of the decreased viscosity^58^. Therefore, it can be concluded that the properties of NADES have a significant influence on the antioxidant activity of the desired molecules.

**Figure 7:**
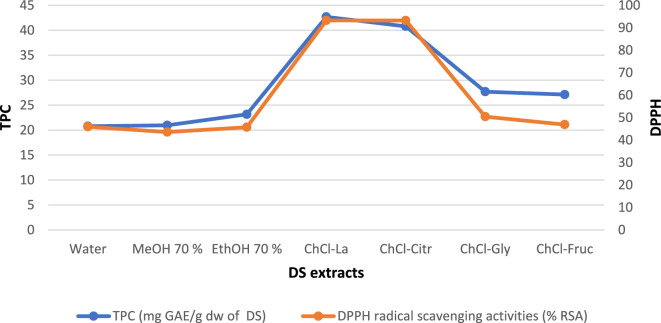
Correlation between total phenolic content and antioxidant activity of date seed extract. MeOH, methanol; TPC, total phenolic content; EthOH, ethanol; La, lactic acid; ChCl, choline chloride; Citri, citric acid; Gly, glycerol; Fruc, fructose; and DPPH, 2,2-diphenyl-1-picrylhydrazyl.

Figure 7 shows a statistically significant, strong, and positive correlation between TPC and DPPH (r^2^ = 0.964). This result was consistent with previous results^1,35,51,59^. Amin and Mukhrizah^60^ found a significant association between the antioxidant activity measured in plant extracts using one assay and the results obtained from other assays. Our results show a statistically significant, strong, and positive correlation between TPC and DPPH. This result was similiter to some previous results^1,35,51,59^. Airouyuwa et al.^36^ reported that The NADES (choline chloride:lactic acid and choline chloride:Xylose) demonstrated the greatest efficacy in extracting bioactive components (TPC) and exhibited the highest antioxidant activity among the tested date seed extracts.

### Analysis of the phenolic composition of DS extracts

**Figure 8:**
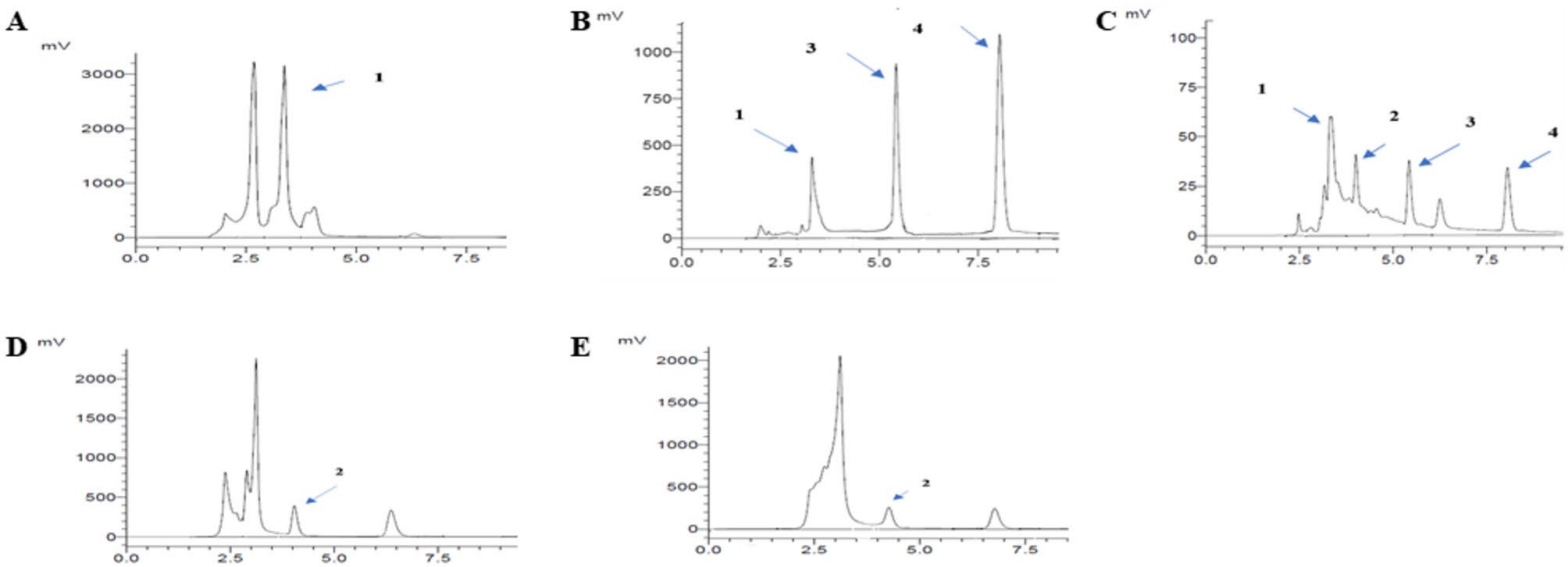
Chromatograms of polyphenols detected at 280 nm in date seeds extracted by: (**A**) water, (**B**) 70% EthOH , (**C**) ChCl–La, (**D**) ChCl–Citri, (**E**) ChCl–Gly, peak (1) gallic acid, (2) catechin, (3) syringic acid (4) *P*-coumaric acid.

**Figure 9:**
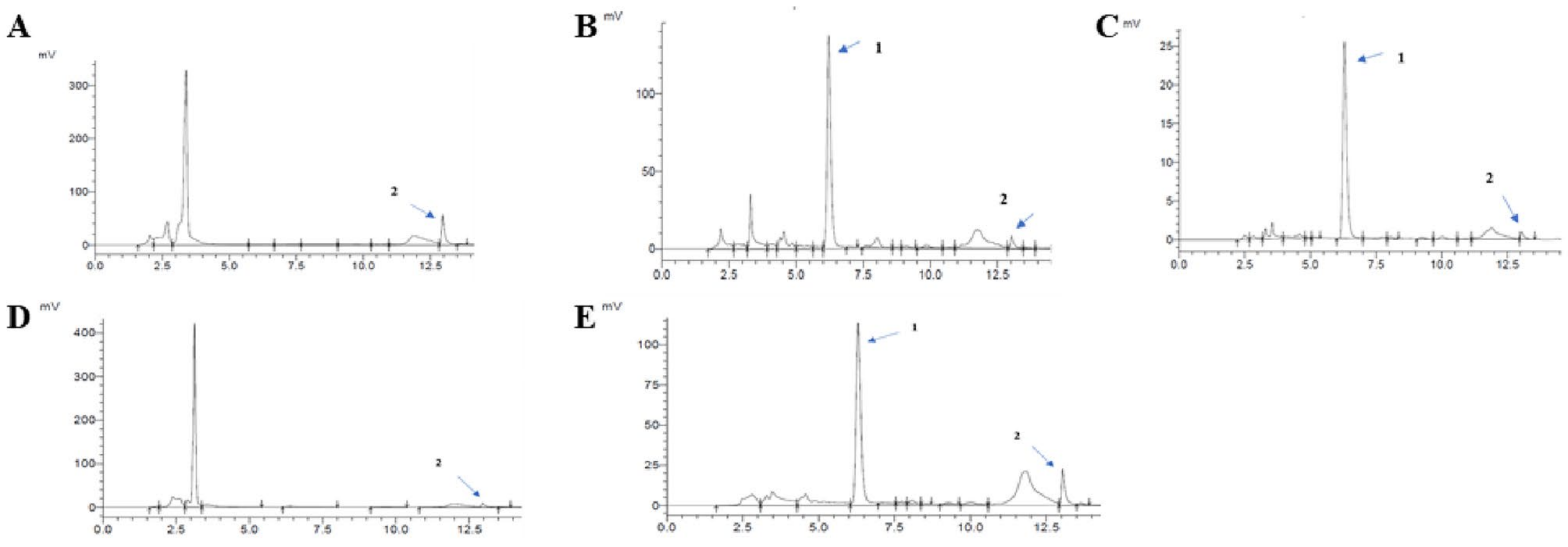
Chromatograms of polyphenols detected at 370 nm in date seeds extracted by: (**A**) water, (**B**) 70% EthOH, (**C**) ChCl–La, (**D**) ChCl–Citri, (**E**) ChCl–Gly. Peak (1) rutin, (2) quercetin.

Table 3 show the concentration by mg/100 g dry weight (dw) of date seeds (DS) for six polyphenols (flavonoids and phenolic acids) in optimal date seeds extracted by three NADES (ChCl–La, ChCl–Citri, ChCl–Gly) and conventional solvents (water, EthOH 70%). Figures 8 and 9 show chromatograms of six phenolic compounds (four detected at 280 nm and tow at 370 nm) in date seeds extracted by three NADES (ChCl–La, ChCl–Citri, ChCl–Gly, DS) and conventional solvents (water, EthOH 70%).

**Table 3:** polyphenols compounds concentration (mg/100 g dry weight of date seeds) in date seeds extracts using natural deep eutectic solvents by HPLC.

Date seeds extracts	Polyphenolic compounds concentration (mg/100 g day wight)					
	Quercetin	Rutin	*p*-coumaric	Syringic acid	Catechin	Gallic acid
Water	9.43 ± 0.04^a^	n.d	n.d	n.d	n.d	860.99 ± 0.01^a^
70% EthOH	4.86 ± 0.00^c,d^	86.01 ± 4.37^a^	128.18 ± 0.73^a^	128.38 ± 2.10^a^	n.d	349.56 ± 15.79^b^
ChCl–La	4.33 ± 0.03^d^	15.33 ± 0.45^c^	2.50 ± 0.28^b^	8.51 ± 0.58^b^	65.49 ± 4.95^c^	46.48 ± 0.68^c^
ChCl–Citri	4.74 ± 0.67^c,d^	n.d	n.d	n.d	502.90 ± 20.48^a^	n.d
ChCl–Gly	6.86 ± 0.55^b^	81.21 ± 1.03^b^	n.d	n.d	419.60 ± 0.56^b^	n.d

EthOH (ethanol); La (lactic acid); ChCl (choline chloride); Citri (citric acid); Gly (glycerol); DS (date seeds); dw (dry weight); n.d (not detected). Different letters indicate significant differences (Duncan’s multiple-range test, *P* < 0.05). The results are mean of duplicate (± SD).

The results indicate that the DS extracted by Water exhibits significantly greater concentrations of gallic acid at 860.99 ± 0.01 mg/100 g of dry weight, compared to other extracts of date seeds. The concentration of catechin was highest in date seeds extracted by ChCl–Citri and ChCl–Gly, whereas no concentrations of catechin were found in the conventional solvent extracts. The concentration of syringic acid and *p*-coumaric acid were found in two extracts, with the highest value observed in the EthOH extract. The concentration of syringic acid and *p*-coumaric acid in DS extracted by EthOH was at 128.38 ± 2.10 and 128.18 ± 0.73 mg/100 g dw respectively.

The finding showed that all DS extracts demonstrated concentration of quercetin. However, water extracts had higher concentrations compared to the other extracts. The rutin had higher concentrations in both DS extracted by EthOH (86.01 ± 4.37 mg/100 g dw) and ChCl–Gly (81.21 ± 1.03 mg/mg dw), than the values in the other extracts. In general, according to the finding gallic acid and catechin were the major identified phenolic compounds in the DS extracts. DS extracts contained minor levels were syringic acid, rutin, and *p*-coumaric acid and quercetin of total phenolic content. Moreover, the results of the quantitative estimation of compounds show that the concentrations of the phenolic compounds in our current study were highest in DS extracted using EthOH. However, concerning identifying compounds, the results showed that DS extracted by ChCl–La recognized all six compounds in the study. Similarly, in a study conducted by Airouyuwa et al.^36^, reported that date seed extract (Khalas variety) using three solvents (80% ethanol, 80% methanol, choline chloride:lactic acid) revealed that the major components were gallic acid and catechin, while rutin, syringic acid, and quercetin were the minor components. In contrast, another investigation examining the ethanol extract of date seeds revealed that rutin and syringic acid are among the major components, whereas gallic and catechins are found in slight quantities^61^.

Date seeds are rich in various polyphenolic compounds, such as rutin, quercetin, catechin, 4-hydroxybenzoic acid, gallic acid, caffeic acid, syringic acid, *p*-coumaric acid, and other chemicals being the most notable among them according to HPLC analysis^20,27,62^, the amounts and types of the polyphenols depend on various factors, including the source and species of the crop^63^, as well as the techniques and solvents employed for extraction^64^, this elucidates the underlying cause for the disparities in high performance liquid chromatography (HPLC) findings across various studies.

The results of our current study show that all phenolic compounds were identified (six out of six) in date seeds extracted by ChCl–La compared to the rest of the extracts. This result is consistent with a analyze conducted by Chanioti and Tzia^1^ identify six out of six phenolic compounds in sample of olive pomace extract using natural deep eutectic solvent formed by choline chloride and lactic acid 1:2 mol ratio, diluted by 20% of water and supported by ultrasound as an extraction method. Moreover, according to Zannou and Koca^38^ four anthocyanins including cyanidin-3-glucoside, cyanidin-3-rutinoside, cyanidin chloride, and pelargonidin-3-glucoside were identified in blackberry extracts obtained by choline chloride and lactic acid (1:2 mol ratio, 20% diluted with water) and ultrasound as an extraction technic. This may be due to their low viscosity in addition to their role as acid-based solvents. ChCl–La is a low-viscosity solvent compared to other NADES^1,38^. NADES is classified as one of the obstacles in dealing with this type of solvent^45^. As it works to limit the transfer of compounds from the solid matrix to the liquid solutions, and thus increases the difficulty of dealing with the decantation, filtration and dissolution processes, as pointed out by Fourmentin et al.^6^ and Jurić et al.^41^.

## Conclusion

Current research provides evidence for the practicality of creating a fast, effective, and environmentally friendly method of extracting natural antioxidants from date seeds. This is achieved by utilizing the NADES as a green solvent along with ultrasound technology. Consequently, this offers a promising approach to using food waste or food by-products sustainably. The study sought a more effective and environmentally friendly strategy for extracting polyphenols from date seeds. The study showed the effectiveness of four NADES (ChCl–La, ChCl–Citr, ChCl–Gly, and ChCl–Fruc), in terms of TPC of DS extracts. The highest value was for ChCl–La and DS-ChCl–Citri extract with values of 42.21 ± 0.90 mg GAE/g dw of DS and 40.35 ± 0.80 mg GAE/g dw of DS respectively. While the lowest values for the total phenol were for DS extracts using conventional solvents with values ranging from 20.57 ± 0.47 to 22.93 ± 0.78 mg GAE/g dw of DS. Moreover, percentage of antioxidant activity (DPPH) of DS extracts derived from carboxylic acids NADES showed higher radical scavenging activities compared to conventional solvents. ChCI–La exhibits the greatest RSA% (93.26 ± 0.13), which is comparable to the RSA% of ChCl–Citri (93.25 ± 0.13). Nevertheless, the identification and quantification of phenolic compounds obtained from date palm seeds using NADES have proven their efficacy as an alternative to traditional solvents.

## Data Availability

The authors confirm that the data supporting the finding of this study are available within the article, all data generated and analysed in this research are available from the corresponding author [Heba A. Sindi] upon reasonable request.
